# Chemical Synthesis and Applications of Colloidal Metal Phosphide Nanocrystals

**DOI:** 10.3389/fchem.2018.00652

**Published:** 2019-01-08

**Authors:** Hui Li, Chao Jia, Xianwei Meng, Hongbo Li

**Affiliations:** ^1^Beijing Key Laboratory of Construction-Tailorable Advanced Functional Materials and Green Applications, School of Materials Science & Engineering, Beijing Institute of Technology, Beijing, China; ^2^Laboratory of Controllable Preparation and Application of Nanomaterials, CAS Key Laboratory of Cryogenics, Technical Institute of Physics and Chemistry, Chinese Academy of Sciences, Beijing, China

**Keywords:** colloidal nanocrystals, indium phosphide, phosphorus precursors, II-V metal phosphide nanocrystals, transition metal phosphides nanocrystals

## Abstract

Colloidal nanocrystals (NCs) have emerged as promising materials in optoelectronic devices and biological imaging application due to their tailorable properties through size, shape, and composition. Among these NCs, metal phosphide is an important class, in parallel with metal chalcogenide. In this review, we summarize the recent progress regarding the chemical synthesis and applications of colloidal metal phosphide NCs. As the most important metal phosphide NCs, indium phosphide (InP) NCs have been intensively investigated because of their low toxicity, wide and tunable emission range from visible to the near-infrared region. Firstly, we give a brief overview of synthetic strategies to InP NCs, highlighting the benefit of employing zinc precursors as reaction additive and the importance of different phosphorus precursors to improve the quality of the InP NCs, in terms of size distribution, quantum yield, colloidal stability, and non-blinking behavior. Next, we discuss additional synthetic techniques to overcome the issues of lattice mismatch in the synthesis of core/shell metal phosphide NCs, by constructing an intermediate layer between core/shell or designing a shell with gradient composition in a radial direction. We also envision future research directions of InP NCs. The chemical synthesis of other metal phosphide NCs, such as II–V metal phosphide NCs (Cd_3_P_2_, Zn_3_P_2_) and transition metal phosphides NCs (Cu_3_P, FeP) is subsequently introduced. We finally discuss the potential applications of colloidal metal phosphide NCs in photovoltaics, light-emitting diodes, and lithium ion battery. An overview of several key applications based on colloidal metal phosphide NCs is provided at the end.

## Introduction

Colloidal nanocrystals (NCs) that exhibit unique optical and electrical properties have attracted considerable attention due to diverse applications such as photovoltaics, optoelectronics, (Coe et al., 2002; Dai et al., 2014; Pietryga et al., 2016; Panfil et al., 2018), and biomedical imaging (Michalet et al., 2005; Hong et al., 2017). Among these NCs, metal phosphide NCs are chemical compounds containing phosphorus and one or more metals, with formula of M_x_P_y_. Phosphorus is known to be able to form at least one stable compounds (e.g., InP, Cu_3_P) with d-group metals and most of the rare earth metals. Besides, many metal phosphides have multiple stoichiometry, providing various crystal structures for binary metal phosphides. Most of the metal phosphide NCs share metal–metalloid bonds (M–P) with a strong covalent component (Greenwood et al., 1966). These bonds are often combined with highly covalent metalloid–metalloid bonds (P–P) (Carenco et al., 2013), where the formation of covalent bond requires harsher reaction conditions for the colloidal chemical synthesis, such as highly active precursors or high reaction temperature. Unlike the metal chalcogenide, metal phosphide can be semiconductors, insulators, and conductors, which are highly dependent on the chemical composition, crystal structure, and electronic state. These specific features provide metal phosphides with unique properties, and also facilitate many potential applications.

Nowadays, conventional cadmium and lead based NCs have been judged because of environmental concerns on heavy metals. Therefore, InP NCs present an attractive alternative owing to low toxicity and emission tunability ranging from visible to near-infrared region (Ramasamy et al., 2017). Zinc phosphide (Zn_3_P_2_) is an intriguing material for photovoltaic (Luber et al., 2013). It has a band gap of 1.5 eV, a large absorption coefficient, a long minority-carrier diffusion length. Transition metal phosphides also have great potential in energy conversion and storage applications.

**Figure 1:**
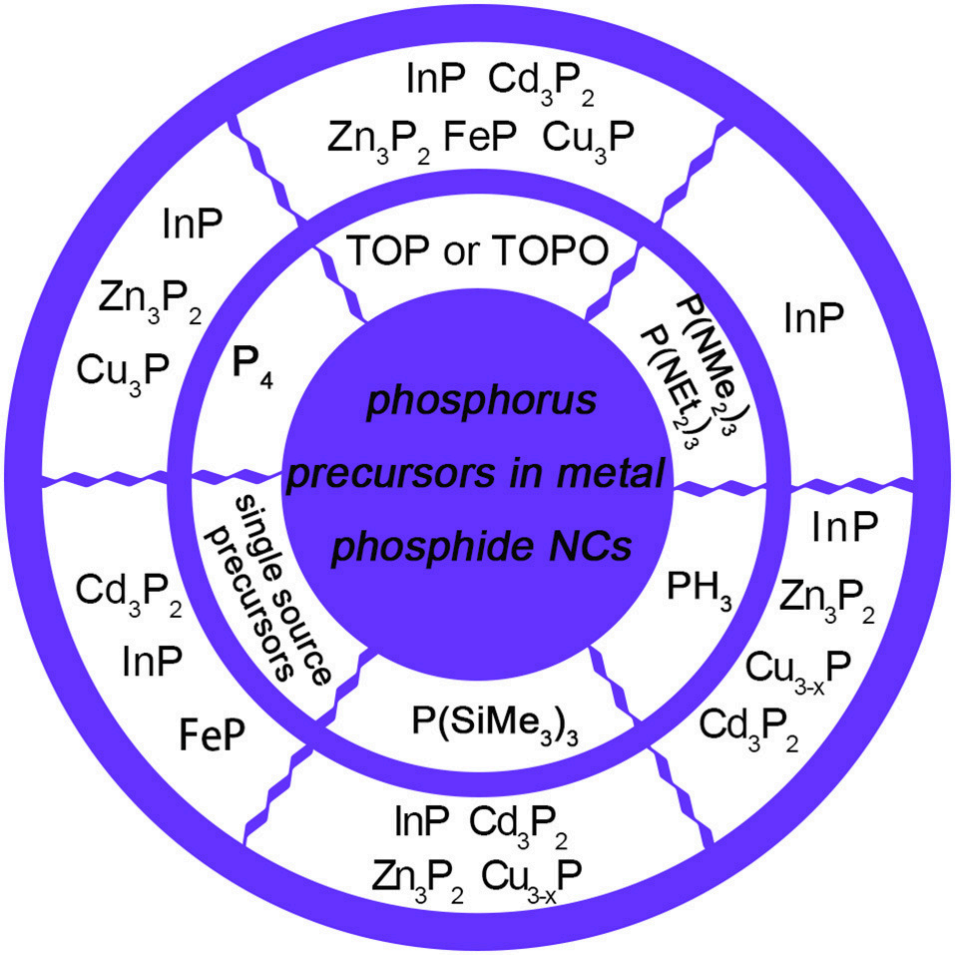
Various types of phosphorus precursors used in the synthesis of metal phosphide NCs. InP NCs could be synthesized by TOP (Lauth et al., 2013), P(NMe_2_)_3_ (Song et al., 2013), P(NEt_2_)_3_ (Tessier et al., 2015), PH_3_ (Li et al., 2008), P(SiMe_3_)_3_ (Micic et al., 1994), P_4_ (Ung et al., 2008), and single source precursors, including In(${\text{PBu}}_{2}^{\text{t}}$)_3_ (Green and O'Brien, 1998) and magic-size clusters (MSCs) (Gary et al., 2015). Zn_3_P_2_ NCs could be synthesized by TOP (Mobarok et al., 2014), PH_3_ (Miao et al., 2013), P(SiMe_3_)_3_ (Ho et al., 2015), and P_4_ (Carenco et al., 2010). Cd_3_P_2_ NCs could be synthesized by TOP (Khanna et al., 2010), PH_3_ (Miao et al., 2012), P(SiMe_3_)_3_ (Miao et al., 2010), and MSCs (Li et al., 2014) acting as single source precursors. FeP could be synthesized by TOP (Henkes and Schaak, 2007) and (CO)_4_Fe(PH_3_) (Hunger et al., 2013) acting as single source precursors. Copper phosphides could be synthesized by TOP (Henkes and Schaak, 2007), PH_3_ (Manna et al., 2013), P(SiMe_3_)_3_ (Liu et al., 2016), and P_4_ (Bichat et al., 2004a).

To synthesize colloidal NCs, cationic, and anionic precursors are indispensable with ligands, non-coordinating solvent as well as high temperature exposure to advance reaction. As for anion precursors in metal phosphide NCs, phosphorus precursors that have been used in the colloidal synthesis can be classified into different types, including the single source precursor (In(${\text{PBu}}_{2}^{\text{t}}$)_3_) (Green and O'Brien, 1998), P(SiMe_3_)_3_, metal phosphorus (Na_3_P) (Jun et al., 2006), magic sized clusters (MSCs) (Friedfeld et al., 2017), elemental phosphorus precursor (Ung et al., 2008; Bang et al., 2017), and PH_3_ gas (Zan and Ren, 2012). Figure 1 shows different types of phosphorus precursors that have been applied in the chemical synthesis of metal phosphide NCs. Regardless of investigations of various phosphorus precursors, P(SiMe_3_)_3_ tend to obtain high quality of InP NCs. However, air and water-free conditions are necessary for the sensitive, hazardous nature of P(SiMe_3_)_3_. In addition, scalability is also a crucial factor because of the demand of large quantity of NCs in commercial applications. In this case, aminophosphine precursors are found to give access to NCs with comparable characteristics as P(SiMe_3_)_3_. In the following sections, the reaction mechanism of InP formation with aminophosphine precursors will be summarized. Several colloidal syntheses utilize TOP and P(SiMe_3_)_3_ to obtain zinc phosphide and transition metal phosphides NCs, which borrow experiences from other metal phosphide NCs (Luber et al., 2013; Ramasamy et al., 2017).

The undercoordinated surface atoms in NC surfaces, acting as trap states, will affect the lifetime, efficiency of electron hole pairs, radiative recombination. Coating an additional shell provides extra means to manipulate the properties. Typically, type I core/shell NCs feature a strongly enhanced photoluminescence quantum yield (PLQY), such as InP/ZnS, and InP/ZnSe. Moreover, insert buffer layers (InP/GaP/ZnS) or adjust the lattice constant of core and shell (InZnP/ZnSeS) can minimize strain and alleviate interfacial defects, which is resulted from lattice mismatch in core/shell NCs.

In this review, a representative introduction on the physical properties of metal phosphide NCs is given firstly. Selecting suitable precursor plays an important role in the chemical synthesis of NCs. Different from the synthesis of metal chalcogenide, much more phosphorus precursors have been tested with the purpose of improving the quality, and reducing the production cost of metal phosphide NCs. We give a detail discussion regarding the underlying chemical mechanism on the phosphorus chemistry in the synthesis of metal phosphide NCs. As one key example of metal phosphide, strategies to obtain high quality InP NCs and their core/shell system with ZnS and ZnSe as passivating shells are summarized. A passivizing shell is of critical importance for InP NCs, because their surfaces are prone to be easily oxidized. We also conclude by pointing out challenges to be addressed in future research, such as broad emission, shape control, and doping of InP NCs. Next, II-V metal phosphide NCs (e.g., Cd_3_P_2_, Zn_3_P_2_) and transition metal phosphides (e.g., copper phosphide, iron phosphide) are presented. Finally, an overview of state-of-the-art applications with metal phosphide NCs is provided, such as light-emitting devices (LEDs), photovoltaic cells, and biomedical applications.

## Phosphorus Precursors in Colloidal Synthesis of InP nanocrystals

As the most important metal phosphide, InP has attracted intensive attention. (Green and O'Brien, 1998; Jun et al., 2006; Ung et al., 2008; Zan and Ren, 2012; Bang et al., 2017; Friedfeld et al., 2017). The direct band gap of 1.35 eV, high fraction of covalent bonding and the large Bohr exciton radius of 9.6 nm make the InP NCs an excellent candidate as visible and near-IR emitting materials. The first liquid colloidal synthesis of InP NCs with narrow size distribution was realized by Nozik in 1994 by using P(SiMe_3_)_3_ (Micic et al., 1994). The P–Si bond in the compound of P(SiMe_3_)_3_ exhibits relatively low dissociation energy (around 363 kJ mol^−1^), which leads to its high reactivity in the case of reacting with an indium precursor. Besides, the phosphorus atom bonded to the highly electropositive Si atom imparts the necessary driving force to react with indium precursors. Therefore, the resulting InP NCs show well-crystallized zinc blende structure and modest exciton peak attributed to InP, which is the first time to show an exciton peak for an III-V NC. This strategy was also applied to the synthesis of other binary and ternary III-V QDs, e.g., GaP. GaInP_2_ (Micic et al., 1995). Since this pioneering report, P(SiMe_3_)_3_ has become the most widely used phosphorus precursor in the colloidal synthesis of metal phosphide NCs. Then, utilizing P(SiMe_3_)_3_ precursor, Micic et al. treated the NCs with a solution of HF or NH_4_F, resulting in highly efficient band-edge emission (Micic et al., 1996, 1997).

In view of their highly reactivity, P(SiMe_3_)_3_ will be consumed in a few seconds. In this context, the growth of NCs tends to proceed via the Ostwald ripening mechanism, which widens the size distribution (Clark et al., 2011). Furthermore, due to pyrophoric nature, high cost, and hazardous reagents of P(SiMe_3_)_3_ and secondary products involved in production, several alternatives for P(SiMe_3_)_3_ are already studied (Reiss et al., 2016). Up to now, following the first synthesis of InP NCs, a variety of phosphorus precursors have been investigated with the main purpose of improving the size distribution. A diversity of phosphorus precursors in the synthesis of InP NCs is summarized in Table 1.

**Table 1:** Summary of phosphorus precursors in colloidal synthesis of InP NCs.

	**P precursors**	**In precursors**	**Methods**	**Results**	**References**
1998	In(${\text{PBu}}_{2}^{\text{t}}$)_3_		Decomposition of In(${\text{PBu}}_{2}^{\text{t}}$)_3_	Quantum confinement effects	Green and O'Brien, 1998
2006	Na_3_P	InCl_3_	Heat-up	Spherical NCs with 5 nm	Jun et al., 2006
2008	P_4_	InCl_3_	Hot-injection	Cubic structure with 3–4 nm	Ung et al., 2008
2008	PCl_3_	In(Ac)_3_	Heat-up	Size tunability; HF-etching improved the quantum yield (QY)	Liu et al., 2008
2008, 2012	PH_3_	In(Ac)_3_	Gas-liquid phase synthesis	InP/ZnS NCs: QY of 30–60%	Li et al., 2008; Zan and Ren, 2012
2012	P(SiMe_2_-tert-Bu)_3_	In(Ac)_3_	Hot-injection	Large size of InP NCs: InP/ZnS NCs: QY of 18–28%	Joung et al., 2012
2012	P(GeMe_3_)_3_	In(MA)_3_	Hot-injection	Improved size distribution	Harris and Bawendi, 2012
2013	P(NMe_2_)_3_	InCl_3_	Hot-injection	Size tunability; InP/ZnS NCs: emission FWHM:60–64 nm; QY: 51–53%	Song et al., 2013
2013	TOP	InCl_3_, InF_3_, InBr_3_	Hot-injection	Size tunability	Lauth et al., 2013
2014	P(SiPh_3_)_3_,P(SiMe_3_)_3_	In(MA)_3_	Hot-injection	Separated the nucleation and growth	Gary et al., 2014
2015	Single-source precursors: InP MA MSCs from P(SiMe_3_)_3_ and In(MA)_3_		Hot-injection	Conversion of InP MSCs to NCs proceeded via a supersaturated solution	Gary et al., 2015
2015	P(NEt_2_)_3_	InCl_3_,InI_3_,InBr_3_	Hot-injection	InP/ZnS NCs: emission FWHM of 46–63 nm; QY: 50–60%	Tessier et al., 2015
2017	P(SiMe_3_)_3_	In(Ac)_3_	Heat-up	Zn–P intermediate complex lowered the reactivity of P(SiMe_3_)_3_	Koh et al., 2017
2017	P_4_	InCl_3_, InI_3_	Hot-injection	Large-scale production; InP/ZnS NCs: emission FWHM: 50–80 nm; QY: 60%	Bang et al., 2017

Similar to P(SiMe_3_)_3_ precursor, single-source precursor is a highly reactive precursor, which can shorten the reaction time and avoid the formation of side products, such as of In_2_O_3_. Mark et al. reported the synthesis of high quality InP NCs by using the single source precursor of (In(${\text{PBu}}_{2}^{\text{t}}$)_3_) (Green and O'Brien, 1998). It is noted that the preparation and storage of such a highly reactive precursor requires great cautions. To address these issues, Li et al. synthesized high quality InP NCs by using the *in situ* generated PH_3_ gas as the phosphorus precursor (Li et al., 2008; Zan and Ren, 2012). PH_3_ is a more stable and safer phosphorus precursor, compared to pyrophoric P(SiMe_3_)_3_ precursor. Furthermore, the method based on PH_3_ gas gives access to larger sized InP NCs. In 2008, Reiss et al. reported the synthesis of InP NCs using an elemental P precursor (red P allotropes) as the phosphorus precursor. To stimulate the reaction, NaBH_4_ was introduced as the reducing agent. The reaction could proceed with a very high reaction yield approaching 100% (Ung et al., 2008). Park et al. reported the synthesis of highly luminescent InP/ZnS NCs using an elemental P precursor of P_4_ (white P) (Bang et al., 2017). They demonstrated that the direct reaction of P_4_ precursor with In precursor without reducing agent. The sublimation of red P powder can simply produce P_4_, therefore P_4_ precursor represents an additional low-cost P precursor and provides an important way to the large-scale synthesis on InP NCs.

In order to avoid the rapid depletion of P(SiMe_3_)_3_, phosphorus precursors with decreased precursor reactivity have been synthesized. Different strategies have been implemented, such as replacing the Si with Ge or Sn (Harris and Bawendi, 2012), substituting methyl group with butyl or aryl group (Joung et al., 2012; Gary et al., 2014). Replacing Si in the Si–P bond with Ge or Sn reduces the reactivity of the precursor by decreasing the polarity of the bond. Likewise, altering the methyl groups to sterically hindering moieties can reduce the reactivity as well. It is believed that using less reactive precursor is helpful to segregate the nucleation and growth and is essential to reach InP NCs with narrow size distribution. Despite great efforts, these alternatives exhibited either largely unchanged or very slowed conversions compared with P(SiMe_3_)_3_.

Aminophosphine presents an alternative phosphorus precursor with advantages of low cost, high stability, decent reactivity, and easy handling (Xie et al., 2007; Laufersky et al., 2018; Mundy et al., 2018). Song and coworkers reported for the first time of high-quality InP NCs using P(NMe_2_)_3_ as a phosphorus precursor (Song et al., 2013). The synthesis was performed by rapid injection of P(NMe_2_)_3_ into the InCl_3_ and ZnCl_2_ solution with oleylamine at 220°C. They found that the size and size distributions of InP NCs are depended on the amount of P(NMe_2_)_3_, ZnCl_2_ as well as the growth temperature and time. The presence of ZnCl_2_ in the synthesis plays a role in activating the aminophosphine to undergo a disproportionation reaction and form P^3−^, which is helpful to achieve narrow size distribution (Laufersky et al., 2018).

**Scheme 1:**
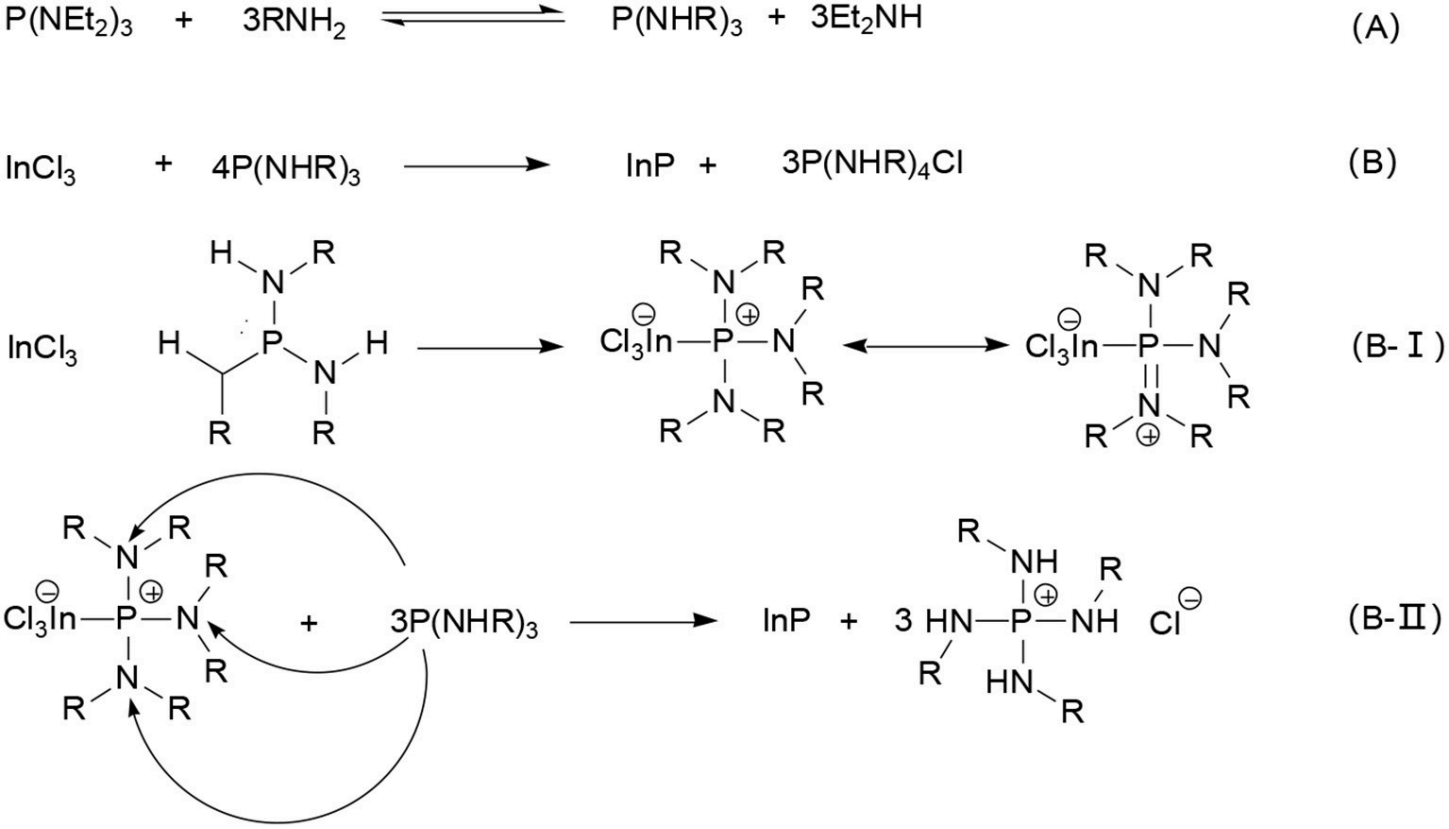
Mechanism of reaction of P(NEt_2_)_3_ with InP NCs **(A)**. P(NEt_2_)_3_ first reacts with the primary amine to obtain P(NHR)_3_, then **(B)**1 equiv of InP is formed by oxidating 3 equiv of the substituted aminophosphine to a phosphonium salt. Namely, **(B-1)** after the synthesis of In-P complex, **(B-II)** P nucleophilic substitution reaction, finally InP unit is obtained. Reproduced with permission from Tessier et al. (2016). © 2016 American Chemical Society.

Later, Tessier presented an investigation into the chemical reactions of InP formation with InCl_3_ and aminophosphine precursors (Tessier et al., 2016). Using NMR spectroscopy and single crystal X-ray diffraction and mass spectrometry, they demonstrated that the InP formation underwent 4P(+III) → P(–III) + 3P(+V) disproportionation reaction. Since aminophosphines are P(III) compounds, reduction steps are needed to form InP with an In (III) precursor. As shown in Scheme 1A, an exchange between primary amines and amino groups of phosphorus precursor occurs, indicating that apart from acting as solvent or ligand, primary amines also play an important role in the whole precursor chemistry. Then, 1 equiv of InP is formed by oxidating 3 equiv of the substituted aminophosphine to a phosphonium salt (Scheme 1B). Namely, substituted aminophosphine has a double role in the reaction, both phosphorus precursor and reducing agent. Based on double role of phosphorus, this mechanism assumes a nucleophilic attack by the phosphorus center of one aminophosphine on an amino group of another aminophosphine. Firstly, InCl_3_ reacts with P(NHR)_3_ to form adducts with different resonance structures (Scheme 1B-I), thus, the positive charge can delocalize over the phosphorus center and the nitrogen atoms, making it possible for residual P(NHR)_3_ to nucleophilic attack nitrogen atoms, as showed in Scheme 1B-II. Subsequent phosphorus nucleophilic substitution results in InP unit formation. This mechanism explains why full conversion of the In precursor is only attained for a 4:1 P/In ratio.

Since then, InP NCs with aminophosphine precursors have been further investigated: utilizing the precursors, the resulting green NCs achieved comparable PL performance to previous work with P(SiMe_3_)_3_ (PLQY: 82 vs. 85%) (Jang et al., 2018). Tetrahedrally shaped InP NCs with improved stability ascribed to oleylamine and chloride ligands (Kim et al., 2016). Zinc salts were also found to activate the aminophosphine precursors by accelerating the formation of the In–P(I) pseudomonomer. Moreover, admixing Cd into a ZnSe shell could also be valid in synthesizing strain-free NCs, suppressing self-absorption, and reducing the amount of NCs for application (Dupont et al., 2017; Rafipoor et al., 2018). Cossairt et al. also demonstrated that aminophosphines were versatile precursors for metal phosphide NCs beyond InP (Mundy et al., 2018).

In short, InP NCs synthesized with aminophosphine precursor, have competitive properties to those from P(SiMe_3_)_3_ routes, indicating that aminophosphine is a promising option for the synthesis of InP NCs. Recently, mechanism of reaction and surface states has been confirmed by X-ray absorption spectroscopy, Raman scattering measurements, and NMR Spectroscopy, etc. (Janke et al., 2018; Laufersky et al., 2018; Tessier et al., 2018). These progresses indicate that synthesis of high quality and efficient InP NCs with the low cost, high stability, decent reactivity, and easy handling precursor will enable to obtain InP NCs on a large scale for a variety of applications, and lead to the extension of colloidal synthesis toward other pnictide NCs.

## Indium Phosphide nanocrystals

InP quantum emitting materials have been considered as an alternative substitution to the conventional quantum dots (QDs), with advantages of less toxicity. Technically speaking, the heavy metal of Cd or Pb in quantum dots-based electronics devices will cause minor environment issue because of the very low concentrations. These heavy metal issues can be further reduced to an even low level by appropriate encapsulation technique and recycling policy after disposal. However, the introduction of heavy-metal materials in electrical and electronic equipment has been restricted the regulation released by Restriction of Hazardous Substances Directive in the EU. Therefore, many researchers have shifted the interests from Cd or Pb based NCs to Cd-free alternatives, i.e., InP QDs.

Indeed, toxicological evaluation reveals that InP QDs display minor toxicity and decent bio-compatibility, thereby suggesting that they can be utilized for potential bio-imaging applications. For instance, Lin et al. found that no observable toxicity *in vivo* after intravenous injection of InP/ZnS QDs in mice for 84 days (Lin et al., 2015). Brunetti et al. studied the toxicity of CdSe/ZnS and InP/ZnS QDs *in vitro* as well as for *in vivo* applications (Brunetti et al., 2013). CdSe/ZnS QDs were shown to induce cell membrane damage, interference with Ca^2+^ homeostasis, which ascribed to the presence of Cd^2+^, while InP/ZnS QDs exhibited low toxicity. Chibli et al. showed that the cytotoxicity induced by the generation of reactive oxygen species was shown to be significantly lower in the case of InP/ZnS QDs as compared to CdTe/ZnS QDs (Chibli et al., 2011). Taken together, toxicity studies demonstrate that these InP based QDs constitute a less toxic alternative to Cd based QDs. All these toxicity evaluations support that the InP QDs pose less environment concerns and guarantee the commercialization of InP QDs based electronics devices.

**Figure 2:**
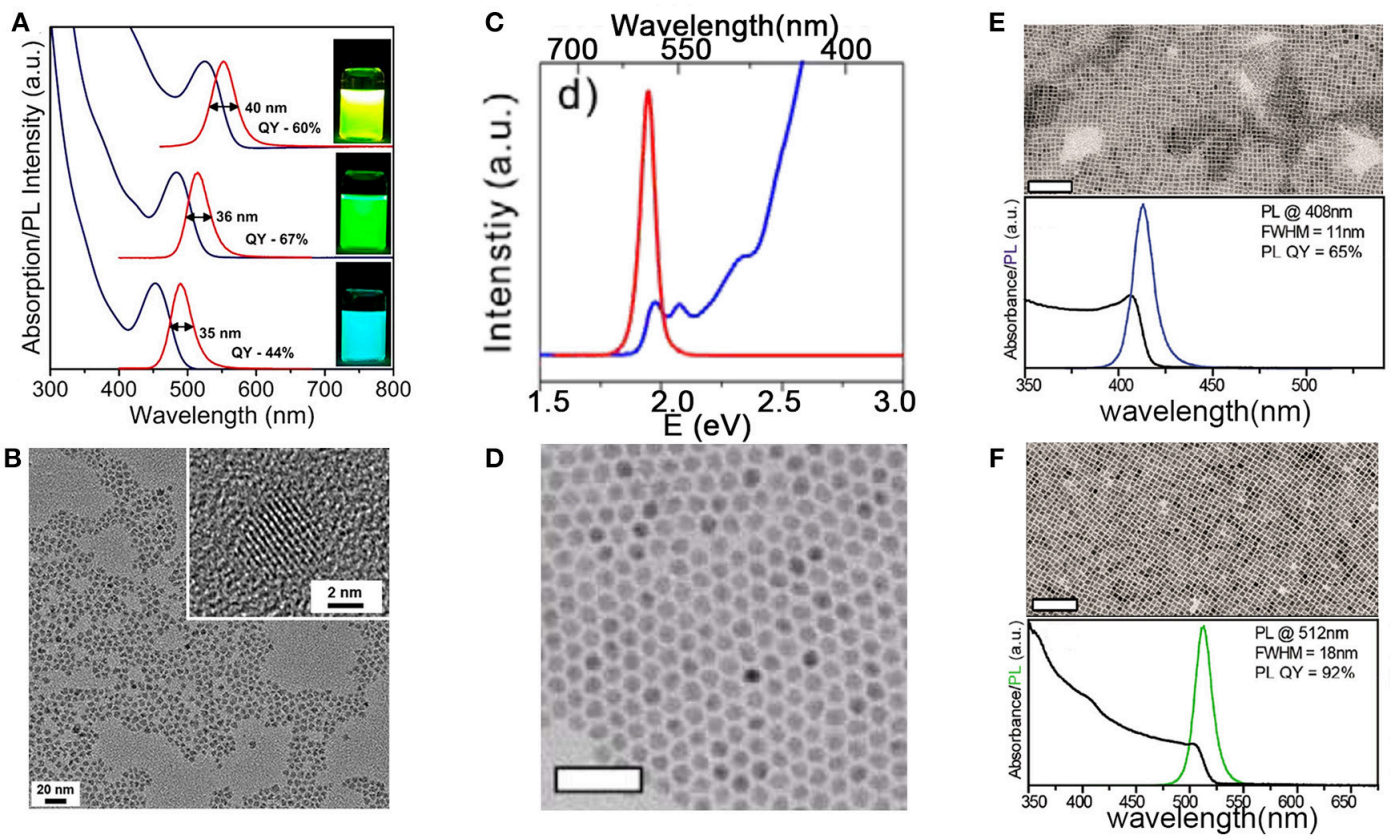
**(A)** In(Zn)P/ZnSe/ZnS NCs emitting at 488, 516, and 552 nm have fwhm of 35, 36, and 40 nm. **(B)** 535 nm emitting In(Zn)P/ZnSe/ZnS NCs with the size of 4.2 nm. Reproduced with permission from Ramasamy et al. (2017). © 2017 American Chemical Society. **(C,D)** Absorption, PL spectra, and TEM images of CdSe/CdS NCs with CdSe core diameters of 5.4 nm and CdS shell thickness of 2.3 nm. Reproduced with permission from Chen et al. (2013) © 2013 Springer Nature. Scale bars are 50 nm. TEM images, absorption, and PL spectra of **(E)** CsPbCl_3_, **(F)** CsPbBr_3_ NCs. Reproduced with permission from Imran et al. (2018). © 2018 American Chemical Society. Scale bars are 100 nm. https://pubs.acs.org/doi/10.1021/jacs.7b13477. Notice:further permissions related to the material excerpted should be directed to the ACS.

At the early stages, InP NCs were characterized as low PLQY, poor photo-stability, in particular its wide PL linewidth, in contrast to Cd-based NCs. In the following decades, the synthesis of InP NCs has achieved progress toward the improvements on the PLQY, PL stability, and narrowing the PL linewidth. Figure 2 shows optical absorption, PL spectra, and TEM images of different NCs. The highest QY in literature for InP QDs was reported above 80%, which is close to unity PLQY of CdSe QDs and cesium lead halide perovskites (CsPbX_3_) NCs (Zhang et al., 2013; Nedelcu et al., 2015). Coating InP NCs with a shell can afford improved photostability, which allows for many applications. However, the wide PL linewidth limits their applications where narrow emission is required, i.e., multiple-color displays. The narrowest PL linewidth, estimated as full-width at half maximum (fwhm), for InP-based core/shell or alloy NCs is 35 nm at 488 nm (Figure 2A) (Ramasamy et al., 2017), which is still inferior to CdSe QDs and CsPbX_3_ QDs.

**Figure 3:**
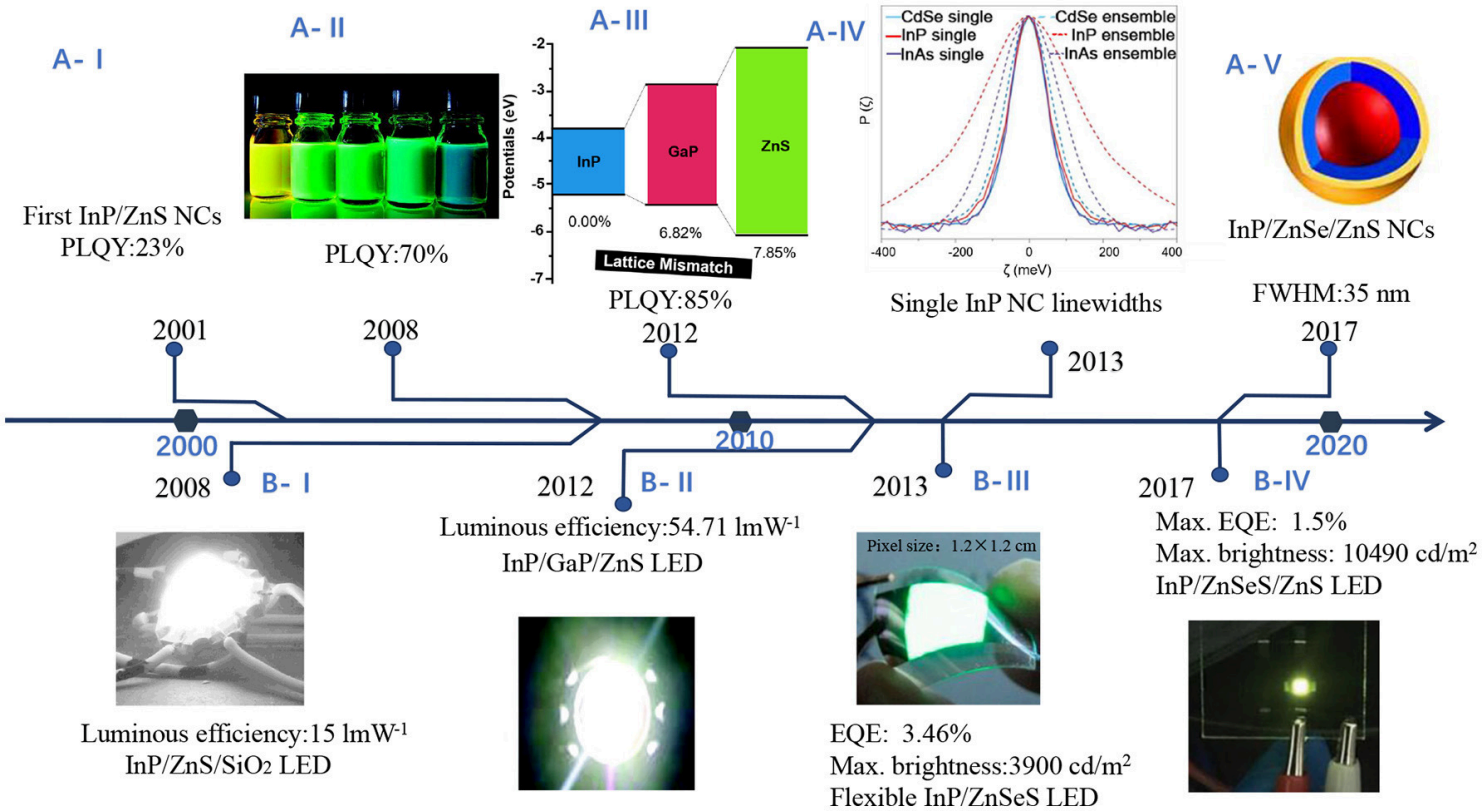
Research timeline illustrating **(A)** improvements on luminescence performance **(B)** progress toward to various applications of InP NCs in light-emitting diodes. **(A-I)** InP/ZnS NCs with PLQY of 23% (Haubold et al., 2001) **(A-II)**. Highly luminescent InP/ZnS NCs. Reproduced with permission from Li and Reiss (2008). © 2008 American Chemical Society. **(A-III)** InP/GaP/ZnS NCs with a maximum PLQY of 85%. Reproduced with permission from Kim et al. (2012) © 2012 American Chemical Society. **(A-IV)** Single InP NC linewidths. Reproduced with permission from Cui et al. (2013) © 2013 Springer Nature. **(A-V)** In(Zn)P/ZnSe/ZnS NCs with emission fwhm as narrow as 35 nm. Reproduced with permission from Ramasamy et al. (2017). © 2017 American Chemical Society. **(B-I)** InP/ZnS NCs as converter material in white LEDs. Reproduced with permission from Ziegler et al. (2008) with permission of John Wiley and Sons. **(B-II)** White LEDs with InP/GaP/ZnS NCs. Reproduced with permission from Kim et al. (2012). © 2012 American Chemical Society. **(B-III)** LEDs with InP/ZnSeS. Reproduced with permission from Lim et al. (2013). © 2013 American Chemical Society. **(B-IV)** LEDs with InP/ZnSeS/ZnS. Reproduced with permission from Wang et al. (2017) with permission of John Wiley and Sons.

In fact, intrinsic emission line width of InP NCs is comparable to CdSe NCs (Figure 3A-IV) (Cui et al., 2013), suggesting that the emission of InP NCs is heterogeneously broadened due to size distribution. The very broad size distribution of InP NCs indicates that separating nucleation and growth is difficult to achieve (Tamang et al., 2016), due to the more covalent nature of the III-V precursors and the fast depletion of precursors at high temperature (Allen et al., 2010).

Several methods have been used to improve size tunability and size distribution, including employing zinc chloride as reaction additive, and replacing P(SiMe_3_)_3_ with lower cost, toxicity, and higher stability phosphorus precursors. Effects of Zn species in InP NC synthesis include the reduction of surface defects, facilitation of subsequent shell growth, and improvement of size distribution (Yang et al., 2012). The use of aminophosphine precursors (P(NMe_2_)_3_ and (P(NEt_2_)_3_) enable the preparation of InP NCs with state-of-the-art quality in the literature.

Increasing core growth time also results in broadened size distribution of InP NCs. Hens et al. expanded this approach by replacing P(NMe_2_)_3_ with P(NEt_2_)_3_, attaining a close to full yield conversion of indium precursor, and demonstrating size tuning was possible by changing the indium halide salt (Tessier et al., 2015). Changing of the precursor concentration is a size-tuning strategy, i.e., higher concentrations lead to smaller NCs, whereas decreasing the concentration appears to deteriorate the size distribution. Inspired by changing the free ligand concentration or the ligand chain length with CdSe NCs (Abe et al., 2013). Tessier and coworkers changed the indium salt halide. When all other reaction conditions were kept constant, the first exciton position can be tuned from 570 to 550 or 520 nm by replacing InCl_3_ by InBr_3_ or InI_3_, respectively, allowing for an efficient size tuning by changing of the indium halide precursor.

Up to now, the narrowest emission of ensemble InP NCs were reported to be ca. 40 nm (Ramasamy et al., 2018), which is still wider than the line widths of PL acquired at the single particle level, suggesting that colloidal synthesis gave rise to the intrinsic inhomogeneity in InP NCs, that was previously attributed to broadened size distribution. Interestingly, recent report reveals that the broad emission may be caused by surface states and lattice disorder, that was confirmed by utilizing X-ray absorption spectroscopy and Raman scattering measurements (Janke et al., 2018). These findings suggest that developing new colloidal synthesis with the purpose of decreasing surface and lattice defects will be the future challenges to achieve comparable performance to CdSe NCs. Furthermore, dopant ions within lattice of NCs were found to be as important as control of the surface states to obtain bright dopant emission (Pu et al., 2017). Ag^+^ dopants can be introduced into lattice of CdSe NCs precisely (Sahu et al., 2012), while few works exist on the doped InP NCs. Recent reports demonstrate that Ag and Cu are of interest as doping agents for dual-emissive InP NCs, therefore, the NCs have potential applications in biological and LEDs applications (Zhang et al., 2015; Yang et al., 2017; Vinokurov et al., 2018). In conclusion, synthetic development specifically for these applications is an interesting avenue to explore.

So far, quite limited progress has been achieved in shape control of InP NCs, only tetrahedral (Kim et al., 2016) and spherical NCs are reported. Colloidal nanoplatelets differ from NCs due to the strong quantum confinement acting in one dimension, which have been most extensively studied. CdSe nanoplatelets can be synthesized with a thickness controlled with monolayer precision, gaining access to a wide spectral range (Christodoulou et al., 2018). Hence, as for InP, development of such 2D heterostructures can be a promising and exciting future direction.

## Indium Phosphide-based Core/shell nanocrystals

The concept of core/shell in NCs has been widely implemented for the conventional CdSe based core/shell structures. Coating an additional shell around the initial seeds provides extra means to manipulate their properties. The obvious benefit is allowing one to easily obtain NCs with enhanced fluorescence efficiency and improved stability against photo-oxidation. This is of particularly importance for the InP NCs, because even freshly synthesized colloidal InP NCs exhibit PLQY of <1% due to surface defects related to P atoms. Talapin et al. found that treating InP NCs with HF can reconstruct the surface and enhance the PLQY up to 40% (Adam et al., 2005). Besides, InP NCs without protecting shell are prone to surface oxidation and photodegradation. Therefore, coating InP with a passivating shell is the prerequisite for further their applications.

Most importantly, band gap offsets and lattice strain between core and shell significantly modify the intraband and interband states, and affect the carrier dynamics of exciton (Dupont et al., 2017; Rafipoor et al., 2018). Therefore, it is possible to prepare the core/shell QDs with unusual and practical photophysical properties. Brainis found that a thick ZnSe shell coating is helpful to reach nearly non-blinking NCs (Chandrasekaran et al., 2017). Hollingsworth et al. reported that coating InP NCs with 11 monolayers of CdS can significantly increase the bi-exciton lifetime up to 7 ns, indicating dramatic suppression of nonradiative Auger recombination (Dennis et al., 2012). So far, ZnSe and ZnS are often used as shell materials for the obvious reason of their large band gap and low toxicity. Herein, we will summarize recent progress toward the synthesis of InP based core/shell direction.

The first issue encountered in the synthesis of core/shell structured InP/ZnSe and InP/ZnS NCs is the relatively large lattices mismatch. In case of former is 3.3%, and in the latter is approaching to 7.7%. The epitaxial growth of shell on top of InP NCs has been proved to be challenging, due to the large lattice constant (a = 5.93 Å). Besides, defects may exist due to the strain at the core/shell interfaces induced by lattice mismatch. Therefore, finding appropriate strategies to avoid defect formation represents one of the main challenges in the synthesis of InP based core/shell NCs.

Overcoating the NCs with a shell of ZnS leads to high PLQY, as shown in Figure 3A. Haubold et al. first passivated the InP core with ZnS, enhanced the PLQY to 23% (Figure 3A-I) (Haubold et al., 2001). They used diethylzinc and bis(trimethylsilyl) sulfide to grow ZnS on InP NCs at 260°C for a limited time, aiming to avoid Ostwald ripening. TEM images and XPS data indicated the formation of ZnS passivated InP NCs. Then one-pot synthesis without precursor injection gave access to InP/ZnS NCs with a maximum PLQY of 70% (Figure 3A-II), narrow emission line width (40–60 nm fwhm) and excellent photostability (Li and Reiss, 2008). The high PLQY is likely attributed to the “smooth” core/shell interface with few defect states. So far, the highest PLQY (85%) (Figure 3A-III) has been reported for the InP/GaP/ZnS NCs with superior photostability as compared to InP/ZnS NCs (Kim et al., 2012), where In^3+^ ions are effectively replaced by Ga^3+^ ions near the surface. The intermediate layer GaP mitigates the effect of lattice mismatch between InP and ZnS. Lattice strain with a ZnS shell may limit a shell thickness <1 nm, making NCs vulnerable against degradable conditions. To alleviate lattice strain, Lee et al. used ZnSe as a lattice adaptor to achieve thicker shells (1.9 nm), the NCs exhibited high PLQY, and enhanced stability under UV irradiation, ligand exchange or rigorous purification (Lim et al., 2011).

**Figure 4:**
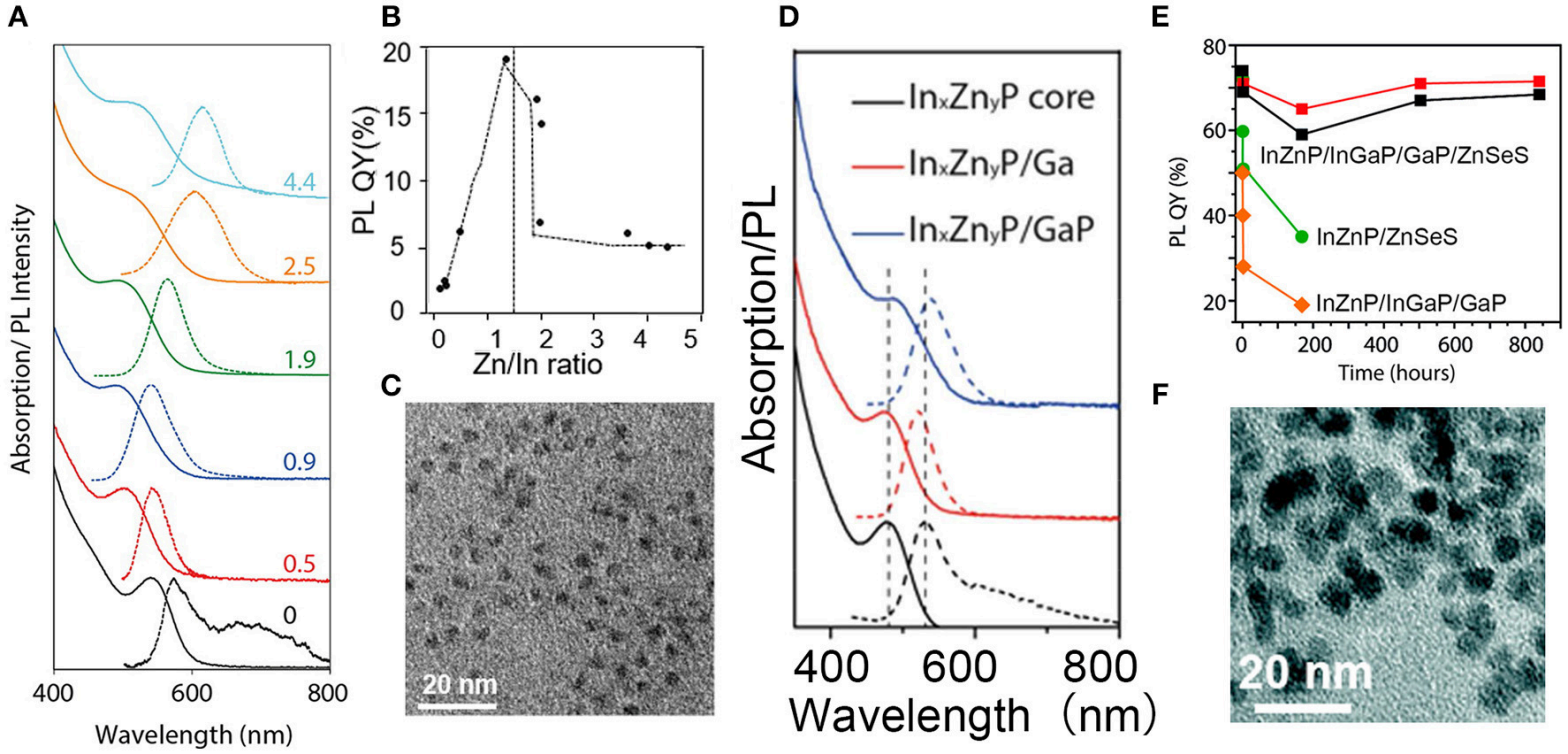
**(A)** Absorption and PL spectra of In_x_Zn_y_P NCs. **(B)** PLQY of In_x_Zn_y_P NCs obtained by Inductively Coupled Plasma (ICP) Optical Emission Spectroscopy (OES) elemental analysis. **(C)** TEM image of In_x_Zn_y_P/ ZnSe_z_S_1−z_ core/shell NCs. Reproduced with permission from Pietra et al. (2016). © 2016 American Chemical Society. **(D)** Absorption and emission spectra of InZnP NCs (Zn/In = 1.5). **(E)** PLQYs for four suspensions of NCs. Four samples were measured: two batches of purified InZnP/InGaP/GaP/ZnSeS NCs (red and black squares), InZnP/ZnSeS NCs (green dots), and InZnP/InGaP/GaP NCs (orange diamonds). **(F)** TEM images of InZnP/InGaP/GaP/ZnSeS NCs. Reproduced with permission from Pietra et al. (2017). © 2017 American Chemical Society. https://pubs.acs.org/doi/abs/10.1021/acs.chemmater.7b00848. Notice:further permissions related to the material excerpted should be directed to the ACS.

Pietra et al. found that In_x_Zn_y_P alloy NCs with a tunable lattice constant can be prepared by changing the amount of zinc precursor, which can match a chosen shell material, possibly creating strain-free core/shell NCs (Pietra et al., 2016). As a result, PLQY of InZnP/ZnSeS NCs can reach as high as 60%. Optical spectra, PLQY and TEM images of In_x_Zn_y_P NCs are shown in Figures 4A–C. They also found that a preferential Ga-for-Zn cation exchange leaded to a InZnP/InGaP core/shell system with increased PLQY when Zn/In ≥0.5. Another GaP and ZnSeS outer shell of InZnP/InGaP NCs exhibit enhanced PLQY (over 70%) and stability (Pietra et al., 2017). Structure and optical spectra of InZnP/InGaP/GaP/ZnSeS NCs are shown in Figures 4D–F.

## Colloidal Synthesis of II–V Metal Phosphide nanocrystals

Cadmium phosphide has been the subject of intensive interest owing to its unique physical properties. Cd_2_P_3_ is a narrow bandgap semiconductor (0.55 eV in bulk) with high dielectric constant (5.8), large Bohr exciton radius (36 nm). All these features suggest that Cd_2_P_3_ in quantum-dot form presents a new material with emission wavelengths span the visible through the near-infrared.

**Figure 5:**
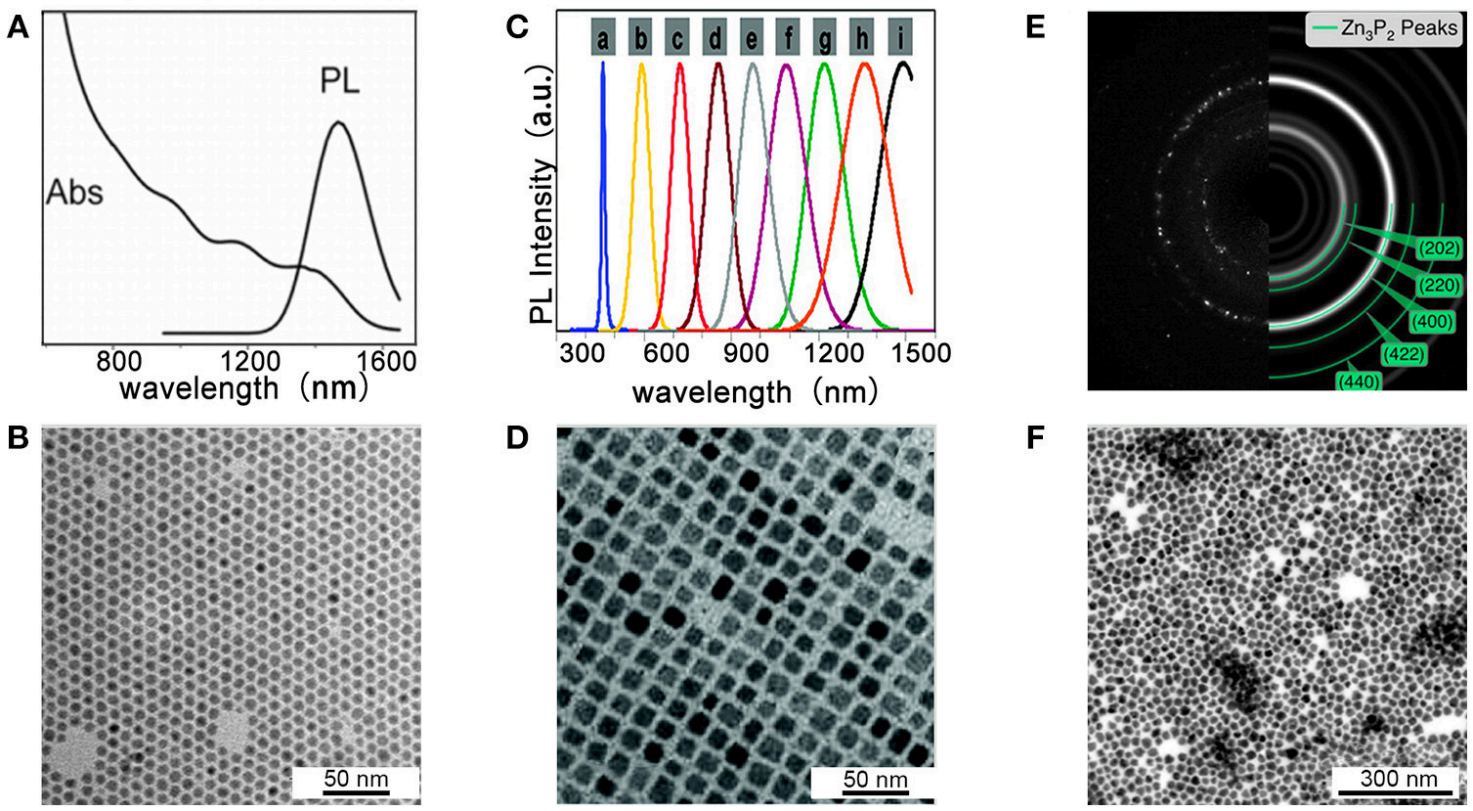
**(A,B)** Cd_6_P_7_ NCs synthesized at 250°C with 40 min growth-time. Reproduced with permission from Miao et al. (2012). © 2012 American Chemical Society. **(A)** Absorption and PL spectra of Cd_6_P_7_ NCs. **(B)** TEM images show high quality of Cd_6_P_7_ NCs. **(C,D)** Cd_3_P_2_ NCs prepared at 250°C with P(SiMe_3_)_3_. Reproduced with permission from Xie et al. (2010). © 2010 American Chemical Society. **(C)** PL spectra of Cd_3_P_2_ NCs with various sizes. **(D)** TEM images of Cd_3_P_2_ NCs with 12 nm **(E,F)**. Zn_3_P_2_ NCs synthesized with P(SiMe_3_)_3_ for 1 h. Reproduced with permission from Mobarok et al. (2014). © 2014 American Chemical Society. **(E)** SAED pattern and simulation of α-Zn_3_P_2_ ring pattern. **(F)** TEM of Zn_3_P_2_ NCs.

Miao et al. for the first time, reported the colloidal synthesis of cadmium phosphide NCs using P(SiMe_3_)_3_ as a phosphorus precursor in the presence of oleylamine and trioctylphosphine as ligands (Miao et al., 2010). By varying the temperature and growth time, they obtained high quality Cd_3_P_2_ NCs with tunable emission ranging from 650 to 1,200 nm, and with high PLQY up to ca. 40%. Further research from the same group found that replacing P(SiMe_3_)_3_ by PH_3_ will reach cadmium phosphide NCs with different stoichiometric (Cd_6_P_7_ instead of Cd_3_P_2_,) and improved size distribution (Miao et al., 2012). Figure 5A shows the absorption spectra with multiple transition of Cd6P7 NCs for the first time. TEM images in Figure 5B show that Cd_6_P_7_ NCs are monodisperse with nearly spherical shape, and the average size of NCs is 6.5 ± 0.3 nm. Since PH_3_ is less reactive compared to P(SiMe_3_)_3_, size of NCs is well controlled by the low reaction rate. This simple and inexpensive strategy paves the way to large-scale production of metal phosphide NCs.

Xie et al. reported an advanced synthesis of Cd_3_P_2_ NCs using P(SiMe_3_)_3_ as a phosphorus precursor and using oleic acid as the only ligand (Xie et al., 2010). They found that under these conditions, the size of the obtained NCs can be tuned in in the range of 1.6–12 nm by simply adjusting the concentration of the oleic acid. Figure 5C shows PL spectra of Cd_3_P_2_ NCs with various sizes, which covers the whole visible and near-IR (450 to over 1,500 nm) region. The optical properties make the NCs useful in optical amplification and lighting. TEM images of Cd_3_P_2_ NCs with 12 nm are given in Figure 5D, where a square array of the NCs is observed.

Cadmium phosphide NCs in the absence of protecting shell can be easily oxidized, which hinders their application in open air. Delpech reported a method for the coating of cadmium phosphide NCs (Ojo et al., 2012). The coating procedure was performed by using zinc acetate and ethylene sulfide as the zinc and sulfur precursors, respectively. They obtained high-quality Cd_3_P_2_/ZnS NCs with PLQY over 50% with modest air stability.

Zinc phosphide (Zn_3_P_2_) has great potential as a solar absorber in thin film photovoltaics, because of its high absorption coefficient in the order of 10^−4^-10^−5^ cm^−1^, large carries diffusion length of 5–10 μm, mostly importantly suitable band gap of 1.5 eV (Green and O'Brien, 2001). The band gap of Zn_3_P_2_ is very close that of CdTe and slightly narrower than CdSe, indicating its high solar absorption capability. Fabrication of Zn_3_P_2_ in the quantum size has been intensively pursued for a long time, because Zn_3_P_2_ NCs can potentially be an emitting material like CdTe and CdSe NCs (Luber et al., 2013). Besides, Zn_3_P_2_ exhibits superior features in contrast to CdTe, CdSe, and InP, because both the Zn and the P are nontoxic and earth abundant elements.

Up to now, there have been several reports on the synthesis of Zn_3_P_2_ NCs. The reported synthetic approaches of colloidal Zn_3_P_2_ NCs borrow experiences from the synthesis of other metal phosphide NCs, utilizing the same phosphorus precursor, such as P_4_, TOP, PH_3_, and P(SiMe_3_)_3_ and the same ligands such as TOP. The zinc precursor includes dimethylzinc, diethylzinc, and zinc stearate. It is worth noting that the formation of Zn_3_P_2_ compounds in liquid solution is difficult compared with other metal phosphides. For instance, significant quantity of ZnCl_2_ is often used, during the synthesis of InP NCs. However, Extensive analysis suggests that under the conditions of the InP synthesis, zincs are not incorporated into the core of the final InP NCs. This feature explains the reason that early reports on the synthesis of NCs usually give poor crystalline and low PL product. This is also consistent with many observations that the synthesis of Zn_3_P_2_ NCs requires much higher temperatures.

Green reported the first colloidal synthesis of Zn_3_P_2_ NCs displaying quantum confined emission (Green and O'Brien, 2001). The synthesis was performed by using dimethylzinc as zinc precursor and the primary phosphine H_2_P^t^Bu as phosphorus source. The obtained particles were revealed as a mixture of both crystalline and amorphous materials. Miao et al. reported the synthesis of Zn_3_P_2_ NCs by using either gas PH_3_ or P(SiMe_3_)_3_ as phosphorus precursor and diethylzinc or zinc stearate as the zinc source (Miao et al., 2013) Buriak and coworkers synthesized monodisperse and pure crystalline Zn_3_P_2_ NCs at low temperature (Mobarok et al., 2014). From the selected area electron diffraction (SAED) pattern in Figure 5E and XRD spectrum, these NCs were found to possess the crystalline tetragonal α-Zn_3_P_2_ structure. TEM image in Figure 5F shows the average size of NCs is 14.7 ± 2.1 nm, with a distribution of 14%. In general, to date the reported synthesis of Zn_3_P_2_ NCs is more limited than cadmium and indium based NCs, mostly likely due to the large differences in reactivity between traditional zinc and cadmium precursors with conventional pnictide sources. Literature on colloidal Zn_3_P_2_ NCs mainly focuses on designing the synthesis and understanding the structure and properties of the NCs instead of their practical device application.

## Colloidal Synthesis of Transition Metal Phosphide nanocrystals

Recently, transition metal phosphide NCs have attracted intense interest for their potential in catalysis, magnetic recording media, and as anode materials in lithium ion batteries (Bichat et al., 2004b; Brock and Senevirathne, 2008). Among these, iron phosphides exist in a wide range of stoichiometry, their properties depend on their physical and electronic structures, such as Fe_3_P and Fe_2_P are ferromagnetic, FeP is super magnetic, FeP_2_ and FeP_4_ are antimagnetic semiconductors (Blanchard et al., 2008). Cu_3_P NCs are air-stable and environmentally friendly materials. When they are used in batteries, their theoretical weight capacitances are slightly higher than that of graphite, and volumetric capacities are more than three times greater than that of graphite (Bichat et al., 2004a).

Nowadays, TOP is often used as a phosphorus precursor in the synthesis of transition metal phosphide NCs. Schaak et al. reported the colloidal synthesis of Cu_3_P NCs for the first time, by injecting the Cu NCs and TOP into the TOPO at high temperature (Henkes and Schaak, 2007). It is found that metals can cause cleavage of the P-C bond, resulting in diffusion of phosphorus into the metal. Falqui et al. optimized the synthesis by replacing Cu NCs with cuprous chloride (CuCl) to react with TOP, and obtained Cu_3_P NCs with improved size distributions (De Trizio et al., 2012). The ratio of TOP and CuCl determined Cu_3_P NCs either by a direct formation of Cu_3_P nucleation or by forming Cu NCs first and then converting into Cu_3_P NCs. The former way leads to reproducible and size controllable pure Cu_3_P, while the latter way results in the formation of Cu-Cu_3_P Janus-like NCs, which is unsuitable for applications in batteries with larger copper domains. Manna et al. reported the synthesis of by using PH_3_ as the P precursor (Manna et al., 2013). The synthesis can be performed under relatively lower reaction temperature. The obtained Cu_3_P nanoplatelets was found to be single crystalline and possess the semiconducting, plasmonic, and rectification properties. More recently, Liu et al. used the more active P(SiMe_3_)_3_ as the P precursor and prepared the Cu_3−x_P NCs at very low reaction temperature (Liu et al., 2016). They further demonstrated that the localized surface plasmon resonance and nonlinear optical absorption of the Cu_3−x_P NCs can be modulated by thermal treatment.

**Figure 6:**
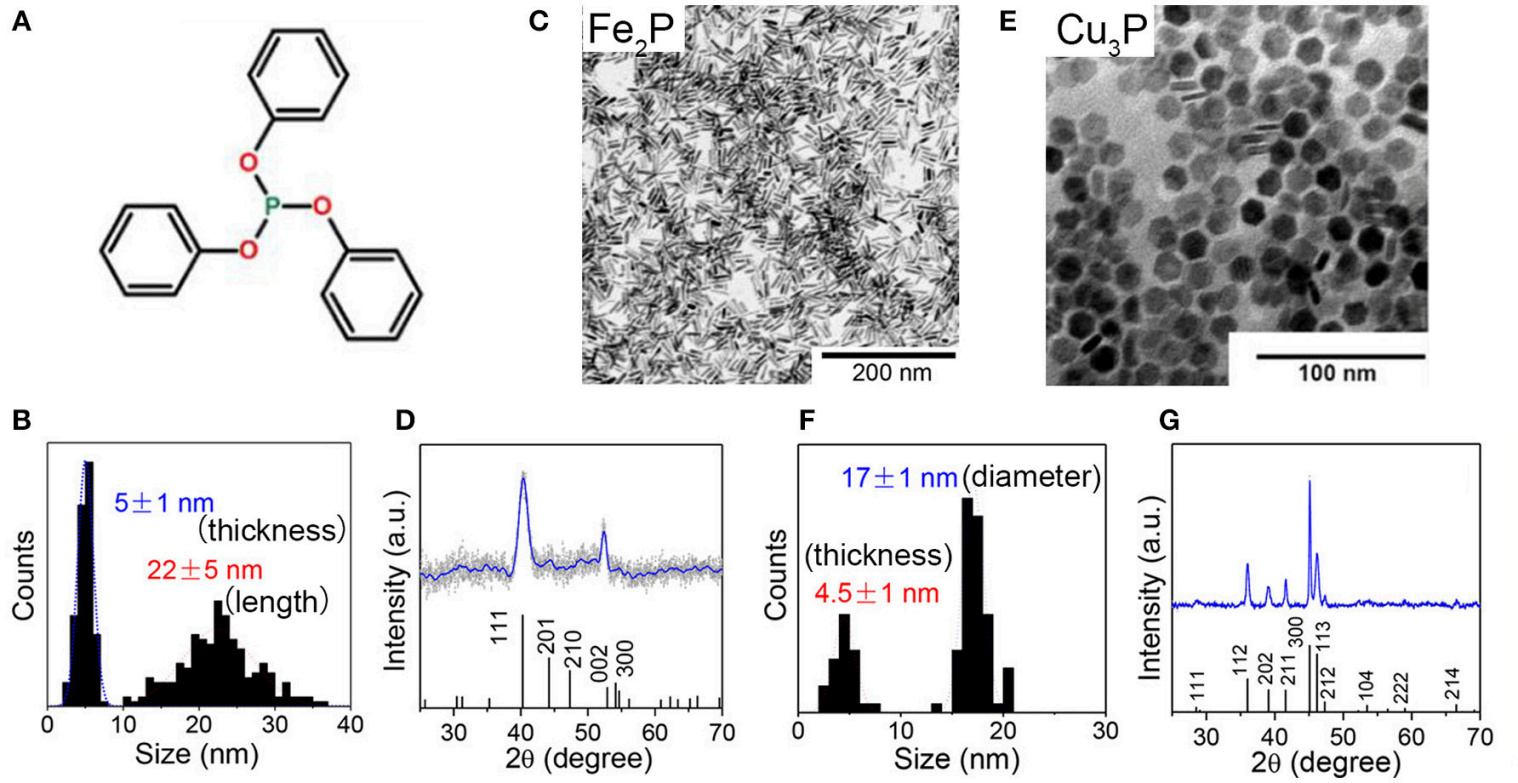
**(A)** Chemical structure of triphenyl phosphite. **(B,C)** TEM images and nanorod length and thickness distribution histograms of Fe_2_P. nanoparticles **(D)** XRD pattern of Fe_2_P nanoparticles. The reference JCPDS pattern of Fe_2_P (01-076-089) is also included in the XRD graph. **(E,F)** TEM images and disk diameter and thickness distribution histograms of Cu_3_P nanoparticles **(G)** XRD pattern of Cu_3_P nanoparticles. The reference JCPDS pattern of Cu_3_P (01-071-2261) is also included in the XRD graph. Reproduced with permission from Liu et al. (2018). © 2018 American Chemical Society.

Perera et al. firstly reported the synthesis of iron phosphides NCs by reaction of Fe(acac)_3_ with P(SiMe_3_)_3_ in TOPO at 260°C, resulting product in pure FeP phase, round shape, narrow size distribution, and high colloidal stability (Perera et al., 2003; Brock and Senevirathne, 2008). Park and coworkers synthesized uniform-sized FeP and Fe_2_P nanorods from thermal decomposition of iron-phosphine complexes (Park et al., 2005). Schaak et al. reported the synthesis of FeP NCs by using pre-synthesized Fe NCs to react with TOP in hexadecylamine (Henkes and Schaak, 2007). Recently, Liu et al. used inexpensive and air-stable triphenyl phosphite (Figure 6A) as the phosphorus sources and prepared rod-shaped Fe_2_P NCs (Liu et al., 2018). Figure 6 displays representative TEM images of the 5 ± 1 nm × 22 ± 5 nm rod-shaped Fe_2_P nanoparticles (Figures 6B,C), 4.5 ± 1 nm × 17 ± 1 nm disk-shaped Cu_3_P nanoparticles (Figures 6E,F).

## Application

Colloidal NCs are covered by a layer of ligands, which can stabilize the colloidal NCs in suitable solvents. This unique feature enables the fabrication of thin film electronic devices by well-established coating and printing techniques. In view of great progress toward the synthesis by means of inexpensive and scalable, wet-chemical synthetic procedures, colloidal QDs are emerging as a versatile class material in electronic devices, in contrast to expensive physically manufacturing processes. Besides, colloidal NCs open up new opportunities to integrate inorganic semiconductors devices with advanced features such as high-performance, large-area, and flexibility.

**Figure 7:**
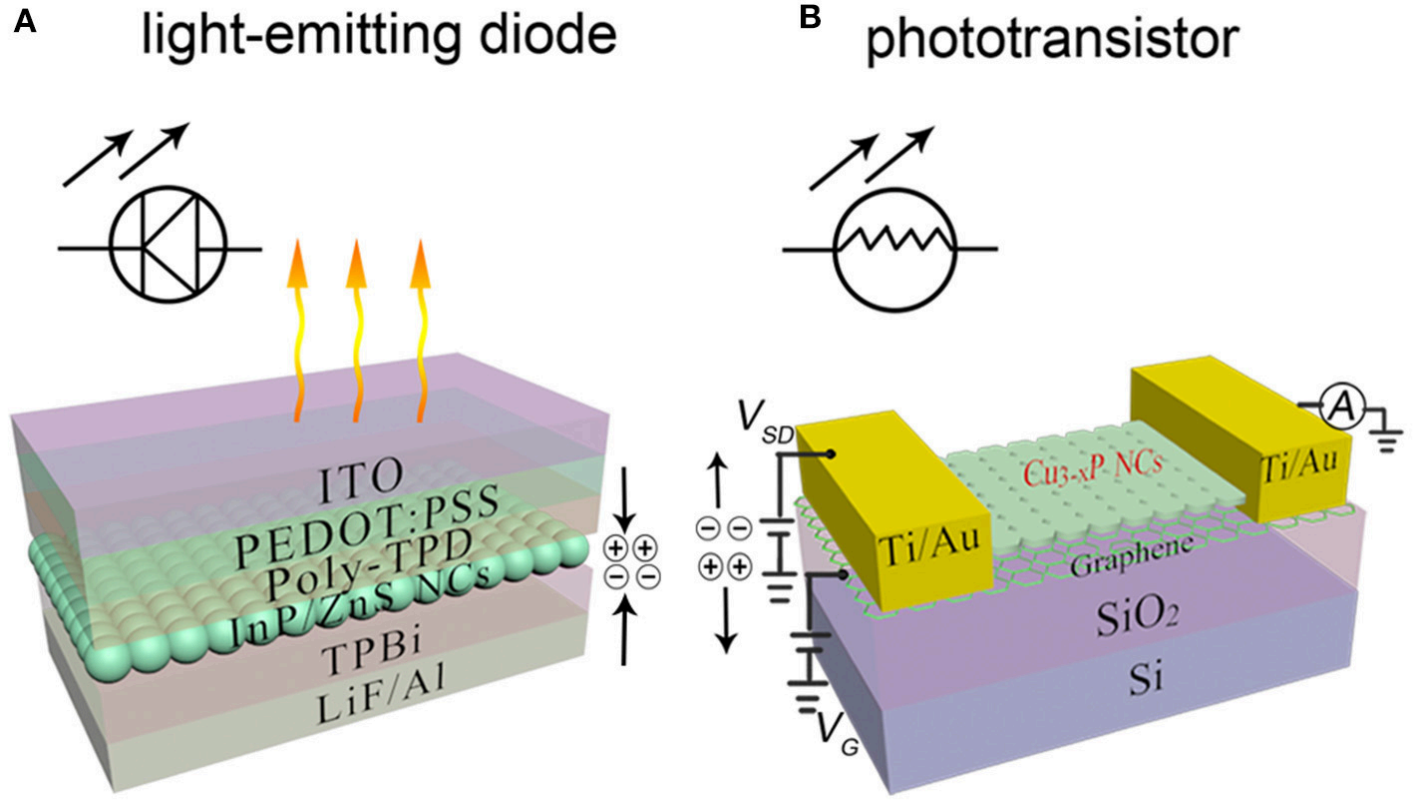
Colloidal nanocrystal device architectures with metal phosphide NCs. **(A)** A white-LED using InP/ZnS NCs. Reproduced with permission from Yang et al. (2012) with permission of John Wiley and Sons. **(B)** Phototransistors based on the hybrid graphene-Cu_3−x_P NC structure. Reproduced with permission from Sun et al. (2017) with permission of John Wiley and Sons.

Among them, colloidal metal phosphide NCs offer a powerful platform for solution-processable electronic and optoelectronic devices, including photodetectors (Keuleyan et al., 2011), photovoltaic cells and light-emission devices (Steckel et al., 2003; Li et al., 2008; Qian et al., 2011), as shown in Figure 7. The actual active element in electronic and optoelectronic devices is macroscopic array of NCs (Talapin et al., 2010), which act as the key components with functions of light absorption or emission and charge transportation (Gaponenko et al., 2016; Kagan et al., 2016). Figure 3B shows progress toward to various applications of InP NCs in LEDs.

Commercialization of conventional quantum dots-based lighting emitting diodes are facing the strict regulation on the using of hazardous substances, such as Cd and Pb. Recent progress toward the synthesis of high-quality InP QDs provides a solution to address this issue. InP QDs have been widely applied in white lighting emitting devices as down conversion phosphor (Ziegler et al., 2008; Yang et al., 2012) and in the framework of electroluminescence devices (Lim et al., 2013; Shen et al., 2017; Wang et al., 2017; Ramasamy et al., 2018). Lim et al. reported design of InP QDs based electroluminescence light emitting diodes (Lim et al., 2013). The device structure based on InP QDs is shown in Figure 7A. By optimizing the charge balance, they observed direct formation of exciton within QDs and efficient radiative exciton recombination. The external quantum efficiency is reported as 3.46% and brightness is 3,900 cd m^−2^. In a recent report, the brightness was improved to 10,000 cd m^−2^ by using ZnMgO as the electron transporting layer to improve the electron injection (Wang et al., 2017). The effect of Auger recombination, Förster resonant energy transfer among the closely packed QDs in a thin film of QLEDs, have a negative effect on device efficiency of QLED. Thus, thick shells or interfacial alloy layer acting as an effective spacer can suppress the above effects. A record of external quantum efficiency of 6.6% in heavy-metal-free red QLEDs have been achieved with a thick ZnS shell of InP/ZnSe/ZnS QDs (15 nm) (Cao et al., 2018). That means there is still more room for improvement and optimization. Moreover, replacing red, green, blue (RGB) color filters with narrow-band green, red InP/ZnSeS/ZnS QD films in color-by-blue QD-emissive liquid-crystal displays (LCDs) results in 1.49 times greater in the relative luminance levels, 2.42 times higher in EQE values than conventional color filter (CF)-LCD, making it possible to replace conventional RGB CF-assisted LCDs (Kang et al., 2018).

Cd_3_P_2_ NCs and Zn_3_P_2_ NCs have been considered as promising materials for photovoltaic applications. Cao et al. proposed PbS/Cd_3_P_2_ quantum heterojunction for NC photovoltaic cells, where p- and n-layers were quantum-tunable and solution-processed infrared light absorbers (Cao et al., 2015). The increased thickness of an n-layer type Cd_3_P_2_ from one layer to two layers resulted in devices exhibiting a power conversion of 1.5%, with an open-circuit voltage of 0.39 V, a short-circuit current of 8.4 mA cm^−2^ and a fill factor of 45%. Luber and coworkers tested photovoltaic performance of heterojunction devices consisting of ITO/ZnO/Zn_3_P_2_/MoO_3_/Ag (Luber et al., 2013). The device possessed excellent rectification behavior (rectification ratio of 600) and photosensitivity (on/off ratio of ~10^2^ under 100 mW cm^−2^ AM 1.5G illumination). Charge carrier mobilities and lifetimes for InP and InZnP QDs are comparable to those of PbS QDs after an (NH_4_)_2_S ligand-exchange procedure, making it possible to utilize In(Zn)P QD films in solar cells. Power conversion efficiencies of 0.65 and 1.2% were achieved of the solar cells based on InP and InZnP QDs, respectively (Crisp et al., 2018).

Recently, Sun demonstrated high-performance, broadband photodetectors with the hybrid graphene-Cu_3−x_P NC structure (Sun et al., 2017). The efficient three-terminal photodetectors (phototransistors) (Figure 7B) exhibit photoresponse from 400 to 1,550 nm with an ultrahigh responsivity (1.59 × 10^5^ A W^−1^) and high photoconductive gain (6.66 × 10^5^) at 405 nm, and a good responsivity of 9.34 A W^−1^ at 1,550 nm. The light-activated functionality of device is provided by a layer of strongly light-absorbing colloidal NCs and an ultrafast carrier transportation graphene layer.

In summary, the prospect of employing metal phosphide NCs in optoelectronic devices offers a number of unique benefits related to their photo-physical properties. Therefore, the colloidal synthesis of various types of metal phosphide NCs has been steadily increasing in recent years and further stimulates their applications. Among them, InP, Cd_3_P_3_, and Zn_3_P_2_ NCs are of significant importance in many light absorption and emission-based applications, such as photovoltaics and light emitting diodes. Particularly, InP and Zn_3_P_3_ are of great interest due to their suitable band gap and low intrinsic toxicity. Despite excellent properties, they are still facing critical issues before they can replace conventional NCs. Compared to chalcogenide-based NCs, metal phosphide NCs exhibit synthetically induced broad size distributions, and low PLQYs and poor thermo- and photo-stability stability. Significant attention has been given to improving these aspects, from viewpoint of chemistry. However, additional attention must be paid to the optimization of their photo-physical properties via structure engineering at the interface and controlling the exciton dynamics. Currently, the performance of optoelectronic devices based on metal phosphide NCs is largely limited due to the undeveloped synthetic methods. Future development of the synthetic approach for metal phosphide NCs is expected to lead to the high-performance optoelectronic devices, i.e., displays.

## Author Contributions

All authors listed have made a substantial, direct and intellectual contribution to the work, and approved it for publication.

### Conflict of Interest Statement

The authors declare that the research was conducted in the absence of any commercial or financial relationships that could be construed as a potential conflict of interest.
